# Impact of cell harvesting methods on detection of cell surface proteins and apoptotic markers

**DOI:** 10.1590/1414-431X202010197

**Published:** 2021-01-08

**Authors:** A. Nowak-Terpiłowska, P. Śledziński, J. Zeyland

**Affiliations:** 1 Department of Biochemistry and Biotechnology, Poznań University of Life Sciences, Poznań, Poland; 2 Department of Genome Engineering, Institute of Bioorganic Chemistry, Polish Academy of Sciences, Poznań, Poland

**Keywords:** Apoptosis, Cell harvesting method, Flow cytometry, Surface antigen

## Abstract

Assays based on the flow cytometry technique allow a convenient analysis of multiple cellular parameters; however, their results should be interpreted cautiously due to a strong impact of confounding factors. Different techniques in cell culturing such as either enzymatic or mechanic detachment of adherent cells can heavily influence the structure of the cell membrane or presence of the surface antigens leading to strong false positive signals, and finally, substantial experimental bias. The aim of our study was to assess and compare the impact of cell harvesting methods (both enzymatic and non-enzymatic) on the apoptosis process and on the surface antigen cytometric analyses. We found significant differences in the quality of analysis in terms of the amount of detected surface markers determined by the detachment method. Our results demonstrated clearly how important it is to carefully choose the appropriate detachment method and may help to avoid mistakes in experiment planning. In conclusion, we recommend to adjust the detachment method to the type of analyzed markers (surface antigens or translocated phosphatidylserine).

## Introduction

Flow cytometry is a technique that allows an efficient assessment of multiple cellular parameters at a single cell level in opposition to bulk methods. The technique is a convenient way of assessing the parameters of cells undergoing apoptosis. It allows a precise examination of effects evoked by, e.g., anticancer drugs. It also allows the study of the expression of the cellular surface proteins, as well as changes in DNA content in cells passing through the cell cycle.

Fluorescence properties of antibodies that specifically bind to epitopes on the surface of cells can be used in cytometric analysis. This type of research allows evaluating whether the protein is present on the cell surface. Information from these studies is particularly important for the assessment of changes occurring on the surface of various types of cells. The level of the fluorescence signal allows assessing whether changes in the level of surface protein expression have occurred during cellular analyses compared with available literature data.

The analysis of the apoptosis process as well as the discrimination between apoptotic versus necrotic cell death is generally based on the changes in cell morphology and on the molecular and biochemical alterations of cells. The most commonly used apoptotic markers are based on changes in the lipid composition and in the integrity of the plasma membrane. Viable cells are characterized by the asymmetry in the plasma membrane phospholipids distribution. Choline phospholipids (phosphatidylcholine, sphingomyelin) are present on the external leaflet of the lipid bilayer whereas aminophospholipids (phosphatidylserine, phosphatidylethanolamine) are present on the inner, cytoplasmic leaflet (1,2 [Bibr B03]). One of the downstream effects in the apoptosis cascade, which is considered a hallmark of apoptosis, is the loss of the plasma membrane asymmetry and, consequently, externalization of phosphatidylserine (PS). Externalized PS exposed at the cell surface serves as a marker for phagocytosis (2–4).

The detection and quantitative measurement of exposed phosphatidylserine is an accurate and reliable assessment of apoptosis incidence and progress. A common means for achieving this is the usage of anticoagulant protein annexin V conjugated to a fluorochrome, e.g., fluorescein isothiocyanate (FITC). The labeled annexin V reversibly binds to PS residues providing a convenient way for apoptotic cells detection. This phenomenon has been widely adopted and exploited in flow cytometry and fluorescence microscopy analyses (1,2).

During the late phases of apoptosis, the integrity of the plasma membrane is reduced and it becomes highly permeable to cationic dyes, such as propidium iodide (PI) or 7-aminoactinomycin D (7-AAD), which serve as a fluorescent marker that intercalates with the major groove of the DNA double helix. Importantly, PI cannot enter the nucleus if the cytoplasmic and nuclear membranes are not already compromised. That phenomenon provides an opportunity to make a distinction between live, apoptotic, and late apoptotic/secondary necrotic cells by using annexin V in conjunction with the plasma membrane permeability marker. Viable cells provide low fluorescence signals both of labeled annexin V and PI (annexin-, PI-). During the early stages of apoptosis, PS becomes accessible for annexin V leading to a strong signal of tagged annexin and still a low signal of PI (annexin+, PI-). At the later stages, cells become intensively stained with both probes (annexin+, PI+). Cells at the end of the apoptotic process, as well as cells with strongly disrupted membranes, may not provide a signal of annexin V but only a high signal of PI (annexin-, PI+).

The assays based on the described method allow a convenient analysis of the apoptotic activity and viability, but their results should be interpreted cautiously due to the strong impact of confounding factors. It has been reported that the different handling procedures, such as either enzymatic or mechanic detachment of adherent cells, transfection, or electroporation, can heavily influence the structure of the cell membrane in terms of PS translocation or permeabilization, leading to strong false positive signals, and finally, substantial experimental bias (2,5).

The commonly used methods of adherent cell detachment include enzymatic and mechanical treatments. A widely used enzymatic method is based on the proteolytic activity of trypsin - an enzyme that cleaves the protein attaching a cell to the flask surface. Another enzymatic method utilizes accutase - an enzyme mixture with proteolytic and collagenolytic activities. Accutase is usually considered a less damaging agent than trypsin and is recommended for the treatment of sensitive cells or for analyses of surface markers. Detachment by the mechanical method is achieved by the use of the so-called ‘rubber policeman’ which is a rubber or plastic scraper attached to a glass rod.

The aim of our study was to assess and compare the impact of cell harvesting methods (both enzymatic and non-enzymatic) on the apoptosis and surface antigen cytometric analyses. We found significant differences in the quality of analysis in terms of the amount of the surface markers determined by the detachment method. Finally, we present the optimal sample preparation procedures for flow cytometry experiments. We recommend adjusting the method to the type of analyzed markers (surface antigens or translocated phosphatidylserine).

## Material and Methods

### Cell lines

Five cell lines were used in this study: two cancer cell lines: MDA-MB-231 (breast adenocarcinoma, ATCC^®^ HTB-26™) and PC-3 (prostate adenocarcinoma, ATCC^®^ CRL-1435™) and three non-cancerous cell lines: MSU-1.1 (fibroblasts), HEK-293 (human embryonic kidney cells), and NT14 (porcine skin fibroblasts). MSU-1.1, HEK-293, and NT-14 were obtained from our own resources. All cell lines were grown in standard growth medium (Dulbecco's modified eagle medium + nutrient mixture F-12, DMEM/F12, Sigma-Aldrich, USA), supplemented with 10% fetal bovine serum (FBS, Sigma-Aldrich) and 1% penicillin-streptomycin (Sigma-Aldrich). All cell lines were grown at 37°C in a humidified atmosphere with 5% CO_2_. Experiments were performed when cells reached full confluence (∼80%).

### Detachment methods

Cells were seeded in 6-well cell culture plates (CELLSTAR) and allowed to attach overnight. After 24 h, when the cells reached 80% confluence, the medium was removed and cells were rinsed with PBS (phosphate-buffered saline, Sigma-Aldrich). Three different harvesting methods were used: two enzymatic methods (trypsinization or treatment with accutase) and one mechanical (scraping with a rubber scraper). For enzymatic methods, cells were incubated with 0.25% trypsin-EDTA solution (trypsin-ethylenediaminetetraacetic acid, Sigma-Aldrich) or with accutase solution (Sigma-Aldrich). Incubation with a given enzyme lasted 10 min for each cell type and was carried out at 37°C in a humidified atmosphere with 5% CO_2_. The dish was not shaken. The detachment was controlled via microscopy.

### Immunofluorescence staining: surface antigen expression of detached cells

To analyze surface antigen expression, detached cells were stained with an antibody against the surface antigen CD55 according to the manufacturers' manual. Cell cultures with full cellular confluence were washed with Hanks' balanced salt solution and detached from the bottom of the vessel using the selected harvesting method. Cells were collected by centrifugation (200 *g* for 10 min at 21°C). For the analysis, cells with a concentration of 1×10^6^/mL were used. The cells were washed with PBS and 0.1% Tween20 and treated with 3% BSA (bovine serum albumin) for 5 min. After washing with PBS and 0.2% Tween20 the cells were incubated with antibody for 45 min at 4°C in the dark (1 μg/mL) to detect the CD55 epitopes on the surface of the cells. Monoclonal antibody (FITC Mouse anti-human CD55, BD Biosciences, own resources) specifically binds to surface antigen CD55, which is also known as complement decay-accelerating factor (DAF).

The wavelength of 520 nm emitted by the excited FITC was quantitatively recorded, which made it possible to analyze the presence of the CD55 epitope on different types of cells. The cells were characterized by two non-fluorescent parameters (forward scatter (FSC) and side scatter (SSC)), and one fluorescent parameter (green fluorescence (FITC detector) from FITC-labeled CD55 monoclonal antibodies). The flow cytometry analyses were performed using the logarithmic gains and specific detectors settings. The threshold was set on the FSC and SSC signals in order to discriminate cells from the cellular debris. The green fluorescence signals from cell populations were displayed on FITC histograms. Each sample was analyzed in triplicate. The determination of CD55 epitope levels on the surface of analyzed cells was based on the median values of fluorescence signals from the FITC detector.

### Apoptosis analysis

Dead cell apoptosis kit with annexin V FITC and PI for flow cytometry (Invitrogen, USA) was used according to the manufacturer's instruction. Harvested cells were washed in cold PBS and resuspended in annexin-binding buffer. The cell density was determined and the suspension was diluted to a final concentration of 10^6^ cells/mL. Five microliters of FITC annexin V and 1 μL of 100 μg/mL PI working solution were added to each 100 μL of cell suspension. The suspension was mixed by gently vortexing and subsequently incubated for 15 min at room temperature in the dark. In the next step, 400 μL of annexin-binding buffer was added, the solution was mixed gently, and then the stained cells were analyzed by flow cytometry using CyFlow^®^ Cube 8 (Sysmex, Germany). Similarly to CD55 analysis, the threshold was set on the FSC and SSC signals in order to discriminate cells from the cellular debris. The wavelengths of 520 nm and 617 nm emitted, respectively, by the excited FITC and the excited PI was quantitatively recorded. The fluorescence signals were displayed on PI-FITC scatter plots. All tests were performed in triplicate.

### Statistical analysis

Data are reported as means±SD. The results were positively tested for normal distribution (Shapiro test) and homogeneity of variance (Bartlett test). For comparison between mean values, ANOVA was used, with Tukey's HSD test for *post hoc* analysis for data with homogeneity of variance. For data without homogeneity of variance, the Dunnett T3 test was used. Values of P<0.05 were considered significant. All statistical analyses were performed using R version 3.4.1 (USA)

## Results

### Effect of different cell-detaching procedures on the cytometric detection of CD55

**Figure 1:**
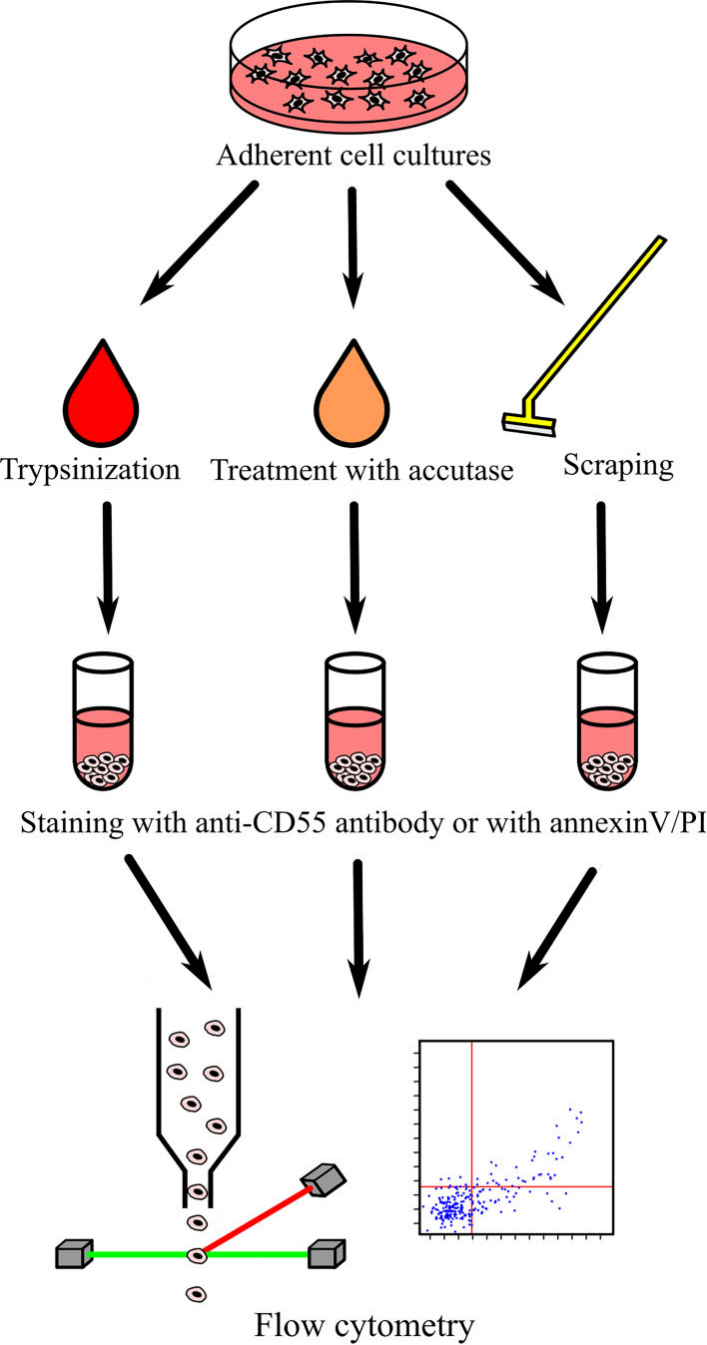
Schematic representation of the conducted experiments.

**Figure 2:**
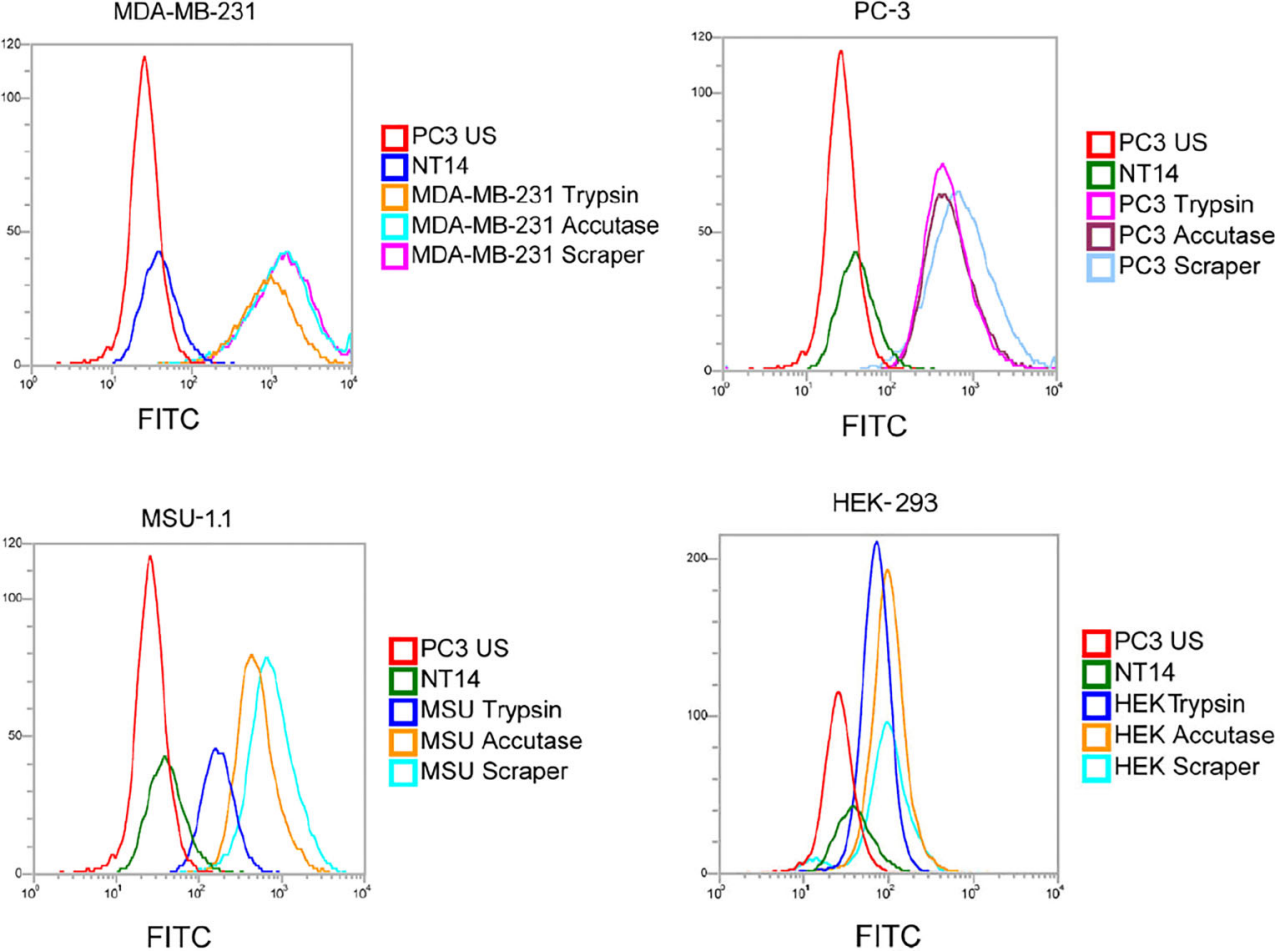
A representative result of flow cytometric analysis of different cell lines using different harvesting methods (trypsin, accutase treatment, scraping by rubber scraper). PC3 US: unstained PC-3 cells (negative control); NT14: wild type porcine cells (no expression of the human CD55, negative control).

**Figure 3:**
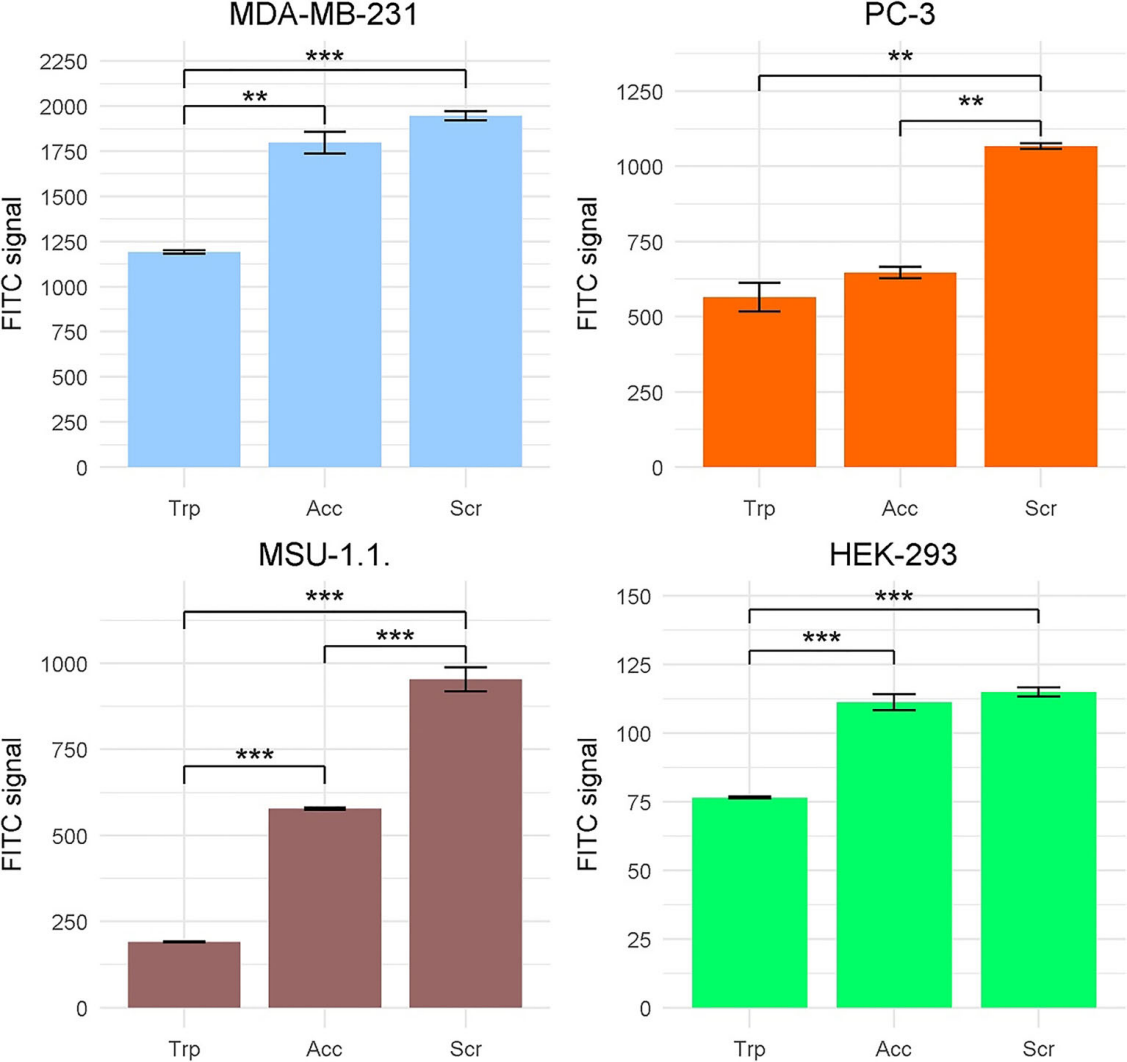
Summarized results of the CD55 level analysis in different cell lines using different harvesting methods [trypsin (Trp), accutase treatment (Acc), scraping by rubber scraper (Scr)]. Data are reported as means±SD. **P<0.01, ***P<0.001 (ANOVA and Tukey's HSD test).

We examined the effects of three different cell-detaching methods on the detection of CD55 surface antigen (Figure 1). Figures 2 and 3 present results of the analyses. We used four cell lines, which naturally express different levels of CD55 irrespectively of the detachment method. Trypsin was the most severe treatment in terms of CD55 presence for all studied cell lines. It led to a significant reduction in the median fluorochrome signal intensity compared to the effects of scraping. The decline of the FITC signal was especially strong in the MSU-1.1 line (Figure 3). The accutase treatment appeared to be significantly less severe than trypsinization; only in PC-3 was no significant difference between trypsin and accutase detected. The mechanical detachment by a rubber scraper lead to the highest level of CD55 antibody signal, however in two lines, MDA-MB-231 and HEK-293, scraping was comparatively effective to the accutase treatment (no significant differences observed).

### Effect of different cell-detaching procedures on the results of apoptosis analyses

**Figure 4:**
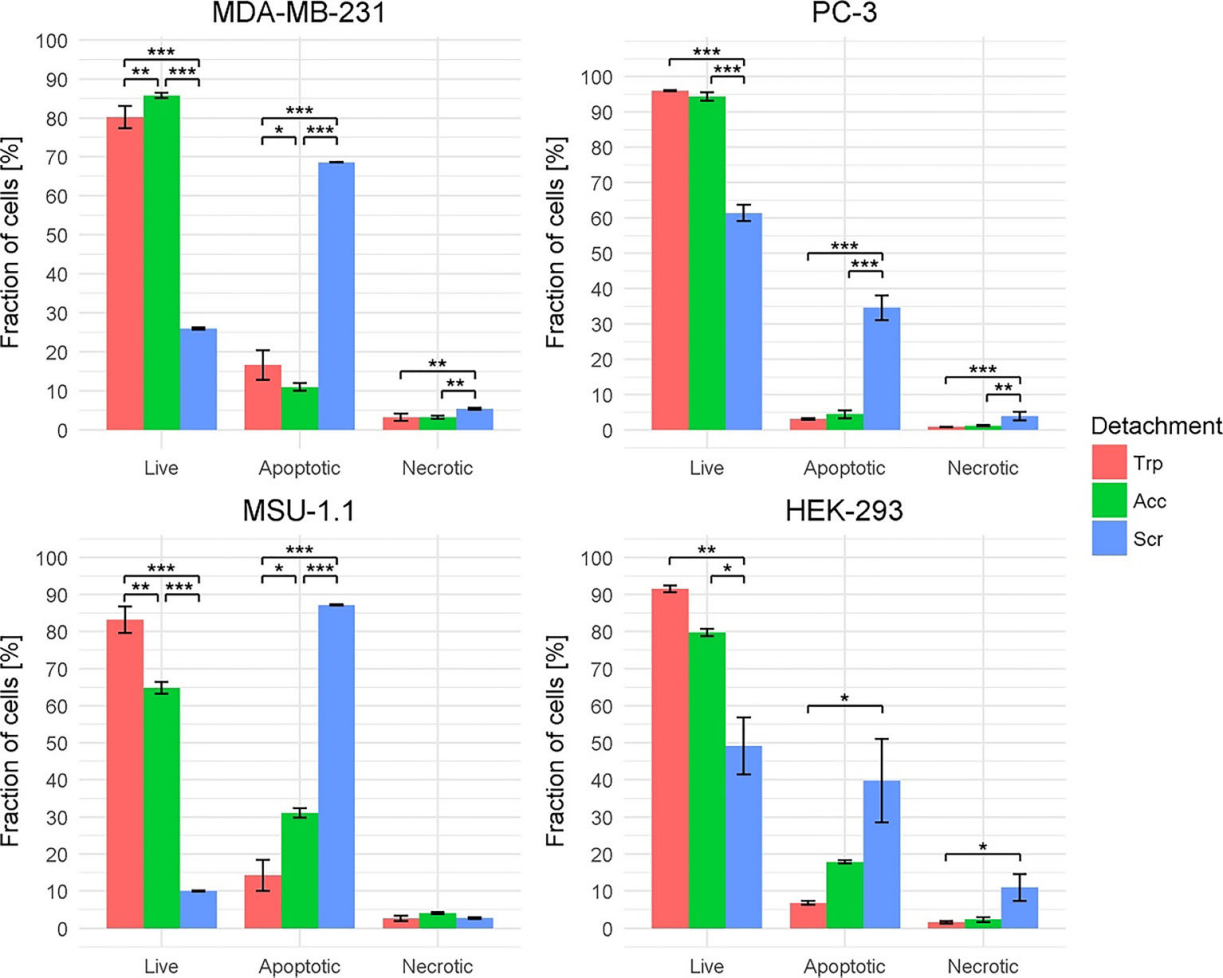
Influence of three cell harvesting methods [trypsin (Trp), accutase treatment (Acc), scraping by rubber scraper (Scr)] on four adherent cell lines. Data are reported as means±SD. *P<0.05, **P<0.01, ***P<0.001 (ANOVA and Tukey's HSD test).

The results of apoptosis analyses are summarized in Figure 4. Among the three tested methods, enzymatic detachment by trypsin and by accutase were the most effective. Trypsinized cells reached the viability level of ≥80.17±2.8% in all tested cell lines. We observed significant differences in viability between the cells treated with trypsin and accutase for two cell lines: MDA-MB-231 (respectively: 80.17±2.8% *vs* 85.7±0.7%) and MSU-1.1 (83.14±3.5% *vs* 64.8±1.6%). The viability of MSU-1.1 cells after the accutase treatment was surprisingly low. The mechanical detachment by a rubber scraper was ineffective - the viability level ranged from 10.04±0.12% in MSU-1.1 to 61.44±2.29% in PC-3.

The percentage of cells labeled as apoptotic ranged from 3.09±0.25% in PC-3 to 31.06±1.3% in MSU-1.1 for enzymatic detachment (either trypsin or accutase). Again, MSU-1.1 cell reaction was surprisingly strong. Mechanical detachment resulted in a high signal of apoptotic cells in all tested lines, ranging from 34.6±3.5% in PC-3 to 87.18±0.09% in MSU-1.1.

The level of necrotic cells ranged from 0.88±0.06% in trypsinized PC-3 to 11.01±3.62% in HEK-293 cells treated with accutase.

The most preferred cell-detaching techniques for all analyzed cell types are summarized in Table 1.

**Table 1:** Most preferred cell-detaching techniques for detecting cell surface proteins and apoptotic markers for different cell lines.

Cell lines	Most preferred cell-detaching procedures for the cytometric detection of CD55
MDA-MB-231	mechanical detachment by a rubber scraper gives a similar result to the accutase treatment for 10 min at 37°C in a humidified atmosphere, with 5% CO_2_ and without shaking
PC-3	mechanical detachment by a rubber scraper
MSU-1.1	mechanical detachment by a rubber scraper
HEK-293	mechanical detachment by a rubber scraper gives a similar result to the accutase treatment for 10 min at 37°C in a humidified atmosphere, with 5% CO_2_ and without shaking

Cell lines	Most preferred cell-detaching procedures for apoptosis analysis
MDA-MB-231	enzymatic detachment by trypsin gives a similar result to the accutase treatment for 10 min at 37°C in a humidified atmosphere, with 5% CO_2_ and without shaking
PC-3	enzymatic detachment by trypsin gives a similar result to the accutase treatment for 10 min at 37°C in a humidified atmosphere, with 5% CO_2_ and without shaking
MSU-1.1	enzymatic detachment by trypsin for 10 min at 37°C in a humidified atmosphere, with 5% CO_2_ and without shaking
HEK-293	enzymatic detachment by trypsin gives a similar result to the accutase treatment for 10 min at 37°C in a humidified atmosphere, with 5% CO_2_ and without shaking

Cell lines: MDA-MB-231: breast adenocarcinoma; PC-3: prostate adenocarcinoma; MSU-1.1: human fibroblasts; HEK-293: human embryo kidney.

## Discussion

In this study, we showed the importance of choosing a suitable cell detachment method for the reliability of flow cytometry results. The appropriate method may vary depending on the type of studied cellular marker.

We used four cell lines commonly used in a variety of studies. MDA-MB-231 and PC-3 are adenocarcinoma cell lines used in, respectively, breast and prostate cancer research. They are widely applied in experiments conducted to evaluate the efficiency of anticancer drugs, including assessment of changes in signaling pathways and apoptotic activity. MSU-1.1 cells represent an immortalized human fibroblast cell line used in studies of malignant transformation. HEK-293 cells are extensively used in cell biology research involving studies of gene expression due to their high susceptibility for transfection. All of these lines naturally exhibit different levels of CD55 expression.

CD55, a complement-regulating protein (decay-accelerating factor, DAF), is a glycosyl phosphatidylinositol (GPI) anchored membrane protein that regulates activation of the immune system. It is found on erythrocytes, lymphocytes, granulocytes, endothelium, and epithelium. CD55 inhibits assembly of the classical and alternative pathway of C3-converting enzymes and protects host cells from the destructive action of autologous complements (6,7). In our study, we analyzed the CD55 antigen, which is considered a representative of other surface proteins, and, therefore, the results obtained in our research of various cell-detaching methods in cytometric detection of surface antigens can be extrapolated to other membrane molecules.

We did not observe any difference between cancerous and non-cancerous cell lines in the susceptibility to membrane damage. The changes in the level of detected CD55 seem to be independent of its natural expression level in a given cell line. However, we analyzed only one surface antigen and a study involving more surface proteins may lead to different results and different conclusions.

Trypsinization by the trypsin-EDTA solution is a widely used enzymatic method of detaching adherent cells from culture flasks. This method exploits the proteolytic activity of a serine protease called trypsin, which cleaves protein related to attachment of cells to the dish surface. It is usually used in combination with the chelating agent ethylenediaminetetraacetic acid (EDTA) due to the role of Ca^2+^ in the process of cell adhesion. Enzymatic detachment, however, can lead to significant changes in cell membrane protein structure and composition leading potentially to significant experimental bias. Mechanical cell harvesting seems to be necessary in that case, e.g., for studying surface proteins expression. On the other hand, mechanical methods can lead to cell membrane damage and to substantial changes in its structure. Thus again, choosing an inappropriate detachment method may cause unintended effects biasing experiment results.

Indeed, Tsuji et al. (8) have demonstrated that trypsinization can lead to a significant decrease in the level of the panel of antigens (including CD55) present in mesenchymal stem cells (MSCs). The authors showed that TrypLE, an animal origin-free, recombinant enzyme, was the most efficient detaching agent since it did not significantly affect the expression level of the studied antigens. The authors have not, however, used any mechanical method.

The study by Bundscherer et al. (5) confirmed that results of annexin V staining can be significantly biased by choosing an inappropriate detaching method in the case of cancer cells. Additionally, they suggested that the observed effects were not dependent on the differentiation grade of the cancer cells or on the tumor entity.

Another example of effects caused by an inadequate harvesting method was shown by Dettmer et al. (9). The authors demonstrated that trypsin/EDTA treatment caused significant metabolite leakage in the SW480 colonic carcinoma cells as opposed to the scraping method.

Many commercially available kits based on annexin V/PI allow a quick and convenient assessment of the apoptotic activity of studied cells. However, they usually do not provide any clear recommendation regarding appropriate cell harvesting methods. This fact can potentially lead to false positive signals and confusion during the results interpretation. Additionally, many authors do not describe the used cell detachment method in their articles. Our results demonstrate clearly how important it is to carefully choose an appropriate detachment method and may help to avoid mistakes in experiment planning.

In conclusion, our results suggest that the enzymatic methods are recommended for the preparation of cells suspension for annexin V/PI apoptosis study. For the analysis of surface antigens expression, on the other hand, mechanical detachment (scraping) seems to be the most reliable method. We recommend, however, to conduct a preliminary experiment to assess the optimal cell harvesting method for every specific case to avoid experimental bias and obtain reliable results.
